# A Importância da Caracterização da Dor Torácica na Conduta em Angina Instável

**DOI:** 10.36660/abc.20240168

**Published:** 2024-04-18

**Authors:** Daniela do Carmo Rassi, Aguinaldo Figueiredo Freitas, Salvador Rassi

**Affiliations:** 1 Faculdade de Medicina da Universidade Federal de Goiás Goiânia GO Brasil Faculdade de Medicina da Universidade Federal de Goiás, Goiânia, GO – Brasil

**Keywords:** Doença Arterial Perférica, Isquemia Miocárdica, Dor no Peito, Síndrome Coronariana Aguda, Eletrocardiografia/métodos

A angina instável é uma entidade clínica de grande complexidade e cujo diagnóstico correto é fundamental para uma conduta apropriada. Esta terminologia, angina instável, tende a entrar em desuso. Como defende a última Diretriz Europeia para Manuseio de Síndrome Coronariana Aguda de 2020,^1^ ela seria englobada sob a denominação de Síndrome Coronariana Aguda (angina instável, infarto agudo do miocárdio com supra e sem supra de ST). Seu diagnóstico é essencialmente clínico, com alterações inespecíficas no eletrocardiograma e sem elevação plasmática de marcadores de necrose miocárdica.^2^

Na Síndrome Coronariana Aguda quando temos alterações eletrocardiográficas e documentação de injúria miocárdica, já há uma conduta estabelecida que seria o cateterismo precoce e a intervenção coronária, se aplicável.^3^ Na ausência destes marcadores, não temos ainda uma conduta estabelecida.

Neste estudo "Avaliação crítica do manejo da angina instável em pronto-socorro terciário de cardiologia" os autores têm como objetivo primário avaliar o manejo da angina instável nesta situação, e como secundário avaliar fatores associados a presença de doença arterial coronariana (DAC) obstrutiva ou isquemia, pelo resultado de exames realizados na estratificação.^4^

Para tanto, analisaram uma coorte retrospectiva de 729 pacientes internados por angina instável, em um período de 20 meses consecutivos, para identificar fatores associados à indicação da estratégia invasiva na angina instável em um banco de dados de um pronto-socorro terciário em cardiologia.

Na avaliação dos fatores associados à estratégia de estratificação, os pacientes foram divididos em forma invasiva (cinecoronariografia) e não invasiva (demais métodos). Para análise de fatores relacionados às alterações nos exames de estratificação, os pacientes foram divididos em grupos com ou sem doença coronariana obstrutiva ou isquemia, conforme exames realizados. Na análise multivariada, estiveram associados à estratificação invasiva, de forma independente, apenas o tabagismo, ex-tabagismo e principalmente dor torácica tipo A. Estiveram associados à doença arterial coronariana obstrutiva ou isquemia, de forma independente, apenas a dor torácica tipo B e a doença arterial coronária prévia.

Estes achados levam os autores a concluírem que o tipo de dor torácica é fundamental não apenas para o diagnóstico da angina instável, mas também na definição do tratamento adequado. Sugerem ainda que estes resultados destacam a importância de incorporar as características da dor aos escores prognósticos endossados pelas diretrizes para otimização do manejo da angina instável. Tanto na Diretriz Brasileira de 2021^5^ como na Diretriz Europeia de 2020,^1^ não há uma enfatização sobre este assunto para as características da dor torácica na escolha do método de estratificação. Os dados apresentados neste estudo evidenciam que o tipo de dor foi a variável mais relevante na escolha da estratégia: pacientes com dor definitivamente anginosa (tipo A) tiveram mais de três vezes chances de ir para o cateterismo cardíaco e não ao uso de escores prognósticos.^6^

Neste estudo chamam a atenção as características clínicas da população estudada, a maioria hipertensos (80%), diabéticos (47%) e portadores de doença arterial coronariana prévia (60%). Não é a população da maioria dos estudos de síndrome coronariana aguda e provavelmente isto se deve às características da instituição, um pronto-socorro aberto de serviço terciário do sistema público de saúde. Outro fato não usual, com relação às características de base dos pacientes, foi que a associação das comorbidades (hipertensão arterial, diabetes, DAC prévia) auxiliassem na indicação de cateterismo cardíaco no cenário da emergência, obviamente por aumentarem a probabilidade de DAC. Entretanto, a única comorbidade mais prevalente no grupo estratificação de risco foi o tabagismo. Ou seja, estes pacientes tinham tendência à estratégia invasiva direta na admissão no pronto-socorro.

Deve-se interpretar com ressalvas o fato de que a presença de DAC prévia, hipertensão arterial e diabetes não influenciaram na escolha do método de estratificação. Uma explicação lógica seria que a alta prevalência destas comorbidades nos pacientes com angina instável deste estudo podem ter enviesado os achados.

Os autores comentam as principais limitações deste estudo, sendo o viés da amostra estudada (centro terciário, único, de alta complexidade) o maior deles, devendo seus achados serem interpretados com cautela para outras populações.

Portanto, a resposta sobre o real benefício da abordagem direta com cateterismo na angina instável ainda permanece incerta.

## References

[1] Collet JP, Thiele H, Barbato E, Barthélémy O, Bauersachs J, Bhatt DL (2021). 2020 ESC Guidelines for the management of acute coronary syndromes in patients presenting without persistent ST-segment elevation. Eu Heart J.

[2] Braunwald E, Morrow DA (2013). Unstable angina: is it time for a réquiem?. Circulation.

[3] Fanning JP, Nyong J, Scott IA, Aroney CN, Walters DL (2016). Routine invasive strategies versus selective invasive strategies for unstable angina and non-ST elevation myocardial infarction in the stent era. Cochrane Database Syst Rev.

[4] Prata MA, Ohe LN, Vilalva KH, Lemos LFN, Smanio PEP (2024). Avaliação Crítica do Manejo da Angina Instável em Pronto-Socorro Terciário de Cardiologia. Arq Bras Cardiol.

[5] Nicolau JC, Feitosa GS, Petriz JL, Furtado RH, Précoma DB, Lemke W (2021). Diretriz da Sociedade Brasileira de Cardiologia sobre Angina Instável e Infarto Agudo do Miocárdio sem Supra desnivelamento do Segmento ST-2021. Arq Bras Cardiol.

[6] Cedro AV, Mota DM, Ohe LN, Timermam A, Costa JR, Castro LS (2021). Associação entre Escores de Risco Clínico (HEART, GRACE, TIMI) e Complexidade Angiográfica na Síndrome Coronária Aguda sem Elevação do Segmento ST. Arq Bras Cardiol.

