# Supplementary material for: A Importância da Caracterização da Dor Torácica na Conduta em Angina Instável
Source: Arq Bras Cardiol. 2024 Apr 18;121(3):e20240168. [Article in Portuguese] doi: 10.36660/abc.20240168 (PMC11081193; doi:10.36660/abc.20240168)
Supplement: Translation

# Translation: The Importance of Characterizing Chest Pain in the Management of Unstable Angina

**Authors:** Daniela do Carmo Rassi, Aguinaldo Figueiredo Freitas, Salvador Rassi

**Affiliations:** 1 Goiânia GO Brazil Faculdade de Medicina da Universidade Federal de Goiás, Goiânia, GO – Brazil

**Keywords:** Peripheral Arterial Disease, Myocardial Ischemia, Chest Pain, Acute Coronary Syndrome, Electrocardiography/methods

Unstable angina is a clinical entity of great complexity and whose correct diagnosis is essential for appropriate management. This terminology, unstable angina, tends to fall into disuse. As advocated by the latest European Guideline for the Management of Acute Coronary Syndrome of 2020,^1^it would be encompassed under the name Acute Coronary Syndrome (unstable angina, acute myocardial infarction with and without ST elevation). Its diagnosis is essentially clinical, with nonspecific changes on the electrocardiogram and without plasma elevation of markers of myocardial necrosis.^2^

In Acute Coronary Syndrome, when we have electrocardiographic changes and documentation of myocardial injury, there is already an established approach that would be early catheterization and coronary intervention, if applicable.^3^In the absence of these markers, we do not yet have an established approach.

In this study "Critical Assessment of the Management of Unstable Angina in a Specialized Cardiology Emergency Room", the authors have as their primary objective to evaluate the management of unstable angina in this situation, and as a secondary objective to evaluate factors associated with the presence of obstructive coronary artery disease (CAD) or ischemia, based on the results of tests performed in stratification.^4^

To this end, they analyzed a retrospective cohort of 729 patients hospitalized for unstable angina, over a period of 20 consecutive months, to identify factors associated with the indication of an invasive strategy in unstable angina in a database from a tertiary cardiology emergency room.

In evaluating the factors associated with the stratification strategy, patients were divided into invasive (coronary angiography) and non-invasive (other methods). To analyze factors related to changes in stratification exams, patients were divided into groups with or without obstructive coronary disease or ischemia, according to the exams performed. In the multivariate analysis, only smoking, ex-smoking, and mainly type A chest pain were independently associated with invasive stratification. Only type B chest pain and previous coronary artery disease were independently associated with obstructive coronary artery disease or ischemia.

These findings lead the authors to conclude that the type of chest pain is fundamental not only for the diagnosis of unstable angina but also in defining appropriate treatment. They further suggest that these results highlight the importance of incorporating pain characteristics into prognostic scores endorsed by guidelines to optimize the management of unstable angina. Both in the Brazilian Guideline of 2021^5^and in the European Guideline of 2020,^1^there is no emphasis on this subject for the characteristics of chest pain when choosing the stratification method. The data presented in this study show that the type of pain was the most relevant variable in choosing the strategy: patients with definitely anginal pain (type A) were more than three times more likely to undergo cardiac catheterization and not to use prognostic scores.^6^

In this study, attention is drawn to the clinical characteristics of the studied population, the majority of whom were hypertensive (80%), diabetic (47%), and had previous coronary artery disease (60%). This is not the population of most acute coronary syndrome studies and this is probably due to the characteristics of the institution, an open tertiary emergency room in the public health system. Another unusual fact, concerning the patients’ basic characteristics, was that the association of comorbidities (hypertension, diabetes, previous CAD) helped in the indication of cardiac catheterization in the emergency setting, obviously because they increased the probability of CAD. However, the single most prevalent comorbidity in the risk stratification group was smoking. In other words, these patients tended to adopt a direct invasive strategy upon admission to the emergency room.

One must interpret with caution the fact that the presence of previous CAD, arterial hypertension, and diabetes did not influence the choice of the stratification method. A logical explanation would be that the high prevalence of these comorbidities in patients with unstable angina in this study may have biased the findings.

The authors comment on the main limitations of this study, with the bias of the sample studied (tertiary, single, highly complex center) being the biggest one, and their findings should be interpreted with caution for other populations.

Therefore, the answer to the real benefit of the direct approach with catheterization in unstable angina still remains uncertain.
